# Association between the frequency of surgeries for video-assisted thoracic surgery and the incidence of consequent surgical site infections: a retrospective observational study based on national surveillance data

**DOI:** 10.1186/s12879-021-06050-6

**Published:** 2021-04-17

**Authors:** Toshiki Kajihara, Koji Yahara, Aki Hirabayashi, Hitomi Kurosu, Motoyuki Sugai, Keigo Shibayama

**Affiliations:** 1 Antimicrobial Resistance Research Center, National Institute of Infectious Diseases, 4-2-1 Aoba-cho Higashimurayama, Tokyo, 189-0002 Japan; 2 Department of Bacteriology II, National Institute of Infectious Diseases, 4-7-1 Gakuen Musashimurayama, Tokyo, 280-0011 Japan

**Keywords:** Surgical site infection, Frequency of surgeries, Video-assisted thoracic surgery, National surveillance, Epidemiology

## Abstract

**Background:**

The association between the frequency of surgeries and the incidence of surgical site infections (SSIs) has been reported for various surgeries. However, no previous study has explored this association among video-assisted thoracic surgeries (VATS). Hence, we aimed to investigate the association between the frequency of surgeries and SSI in video-assisted thoracic surgeries.

**Methods:**

We analyzed the data of 26,878 thoracic surgeries, including 21,154 VATS, which were collected during a national surveillance in Japan between 2014 and 2018. The frequency of surgeries per hospital department was categorized into low (< 50/year), moderate (50–100/ year), and high (> 100/year). Chi-squared test or Fisher’s exact test was used for discrete explanatory variables, whereas Wilcoxon’s rank-sum test or Kruskal-Wallis test was used for continuous explanatory variables. Univariate analysis of the department groups was conducted to explore confounding factors associated with both SSIs and the department groups. We used a multiple logistic regression model focusing on VATS and stratified by the National Nosocomial Infections Surveillance System (NNIS) risk index.

**Results:**

The rates of SSIs in the hospital groups with low, moderate, and high frequency of surgeries were 1.39, 1.05, and 1.28%, respectively. In the NNIS risk index 1 stratum, the incidence of SSIs was significantly lower in the moderate-frequency of surgeries group than that in the other groups (odds ratio [OR]: vs. low-frequency of surgeries: 2.48 [95% confidence interval [CI]: 1.20–5.13], *P* = 0.0143; vs. high-frequency of surgeries: 2.43 [95% CI: 1.44–4.11], *P* = 0.0009). In the stratum of NNIS risk indices 2 and 3, the incidence of SSI was significantly higher in the low-frequency of surgeries group (OR: 4.83, 95% CI: 1.47–15.93; *P* = 0.0095).

**Conclusion:**

The result suggests that for departments with low-frequency of surgeries, an increase in the frequency of surgeries to > 50 per department annually potentially leads to a decrease in the incidence of SSIs. This occurs through an increase in the experience of the departmental surgeons and contributes to the improvement of VATS outcomes in thoracic surgeries.

**Supplementary Information:**

The online version contains supplementary material available at 10.1186/s12879-021-06050-6.

## Background

Thoracic surgery (THOR) is a standard modality for early non-small cell lung cancer and recurrent pneumothorax. Video-assisted thoracic surgery (VATS) has been widely used to treat lung cancer and pneumothorax in the past two decades because of its minimally invasive technique [1]. In Japan, in 2018, the number of lung cancer operations using VATS was 34,249 and accounted for 75.7% of the total operations (*n* = 45,243), whereas that of pneumothorax-related operations using VATS was 14,379 and accounted for 97.6% of the total operations (*n* = 14,731) [2].

Surgical site infections (SSIs) remain among the most frequent healthcare-associated infections worldwide [3]. In the European point prevalence survey between 2016 and 2017, the incidence of SSIs was 18.3% [4], and the estimated number of related deaths per year was 16,049 in the European Union [5]. The cost of healthcare-related infections amounted to US $17,916 for colon surgeries and US $34,741 for coronary artery bypass graft surgeries [6]. In this context, preventing SSI not only leads to good patient outcome, but also has economic benefits [7]. In thoracic surgery, SSIs are also associated with increased morbidity, in-hospital mortality, prolonged hospitalization, and increased costs [8]. There are various reports of risk factors for SSI, for example, age, sex, smoking, body mass index, duration, American Society of Anesthesiology score, wound class, emergency operation, which depend on the types of surgeries performed [9]. VATS has improved the incidence of postoperative complications, and thus, has been increasingly used in thoracic surgeries [10].

The association between the frequency of surgeries per department and the incidence of SSI in various surgeries is yet to be clarified because findings have been conflicting. As an influencing factor of the incidence of SSI, a previous study showed that surgeons with a higher frequency of surgeries (i.e., surgeons with a large number of surgeries conducted per period) tended to be associated with a lower incidence rate of SSIs than their colleagues with a lower frequency of surgeries [11, 12]. Further, departments with a higher frequency of surgeries were associated with better patient outcomes [13–15]. In contrast, Furuya-Kanamori et al. reported that groups with high-frequency of surgeries tended to have a higher risk of encountering SSIs than groups with low-frequency of surgeries in colorectal surgeries [16]. Specifically, there has been no study focusing on video-assisted thoracoscopic surgery (VATS). The association between the frequency of VATS and the incidence rate of SSIs remains unknown.

To investigate the association between the frequency of surgeries and SSIs in video-assisted thoracic surgeries, we utilized a subset of the national SSI surveillance data collected in Japan, where surveillance for SSIs has been conducted since 2002 as the Japan Nosocomial Infections Surveillance (JANIS) [17, 18], which is one of the largest SSI databases.

## Methods

### Study design and data source

This retrospective observational study extracted the surveillance data of patients who underwent THOR between 2014 and 2018, from the JANIS database. Briefly, the JANIS database includes data on 305,960 surgeries from 802 institutions in 2018. The SSI surveillance in JANIS is conducted using the definition of the US National Nosocomial Infections Surveillance System (NNIS), with some modifications. It is comparable to other international SSI surveillances, such as the OP-KISS of Germany [19] and the National Healthcare Safety Network of the United States of America [20]. THOR data using only cardiac or cardiovascular surgeries were not included in the JANIS database. Patient information is de-identified by each hospital before submission to the JANIS database. Hospitals are recruited on a voluntary basis each year for the JANIS, and the participating hospitals are required to report SSI surveillance data for selected operative procedures electronically on a biannual basis [21].

### Data collection

In total, 247 departments across 247 hospitals (each hospital has one thoracic surgery department) participated in the JANIS SSI surveillance for at least 1 year from 2014 to 2018. They submitted the data of 39,368 THOR procedures during the period. The data of 26,878 cases of THOR performed in 74 hospitals, which completed data submission during the 5 years (from 2014 to 2018), were included in the present study.

The variables analyzed included NNIS risk index, age, sex, multiple procedures, emergency operation, implant, trauma surgery, general anesthesia, and video-assisted surgery. The United States Center for Disease Control defines the NNIS risk index as the sum of three binary variables indicating whether 1) the American Society of Anesthesiologist score is higher than two, 2) wound classification is contaminated or dirty, and 3) duration of the procedure in minutes is longer than the 75th percentile. It is a convenient risk stratification system widely used worldwide, including in the JANIS [22]. The departments were classified into three department groups according to the frequency of surgeries (i.e., the average number of procedures per department per year) as low (< 50/year), moderate (50–100/year), and high (> 100/year) based on its distribution (Additional Fig. 1) according to a previous study [23].

### Statistical analysis

Potential risk factors for SSI were first explored using univariate analysis. Chi-squared test or Fisher’s exact test were used for discrete explanatory variables, whereas Wilcoxon’s rank-sum test or Kruskal-Wallis test were used for continuous explanatory variables. A univariate group analysis of the department groups was conducted to explore confounding factors associated with both SSIs and the department groups. Multiple logistic regression analysis was performed to estimate the odds ratios (ORs) and 95% confidence intervals (CIs) of the risk factors after adjusting for confounding factors. We fitted the multiple logistic regression model stratified by the NNIS risk index after grouping risk indices 2 and 3 into a stratum for clear interpretation. The stratification was conducted using the NNIS risk index rather than the three binary variables comprising it because they were highly correlated with each other: variable 2) was highly significantly associated with variables 1) and 3) (*p* < 0.001), and there was a significant association between variables 1) and 3) (*p* = 0.015). All statistical analyses were performed using JMP Version 13.2.1 (SAS Institute, Cary, NC, USA). A *P* value < 0.05 was considered statistically significant.

### Ethical considerations

Patient identifiers were de-identified by each hospital before data submission to JANIS. The anonymous data stored in the JANIS database were exported and analyzed.

The protocol of this study was approved by the Ministry of Health, Labor and Welfare (approval number 0417–1) according to Article 32 of the Statistics Act and in accordance with the Helsinki Declaration. The requirement for informed consent was waived by the Ministry of Health, Labor and Welfare (approval number 0417–1).

## Results

### Total incidence of SSIs in the THOR procedures and their characteristics

The total number and rate of SSIs in the THOR procedures during the study period were 335 and 1.25%, respectively (Table 1). A total of 21,154 VATS procedures were performed, accounting for 78.7% of all THOR procedures. The number of VATS procedures was approximately four times greater than that of open thoracic surgeries. There were 17,615 (65.5%) male patients. The most dominant SSI was organ/space SSI (48.4%), followed by superficial incisional infections (38.2%). Causative pathogens were identified in 59.4% of all cases, with the most common being methicillin-resistant *Staphylococcus aureus* infection (64 isolates), followed by methicillin-susceptible *S. aureus* infection (54 isolates). The most common causative gram-negative bacterium was *Pseudomonas aeruginosa* (14 isolates). Polymicrobial infections were found in 30 cases.

**Table 1:** Descriptive statistics of the procedures, patients, and SSI

Total no. of procedures (from 2014 to 2018) among the 74 departments	26,878	
Video-assisted thoracic surgery	21,154	
Open thoracic surgery	5724	
Emergency surgery	1569	
Mean age	62.1	(0–97)
Male sex (%)	17,615	(65.5)
Incidence of SSI, N (%)	335	(1.25)
Type of SSI, N (%)		
Superficial incisional	128	(38.2)
Deep incisional	45	(13.4)
Organ/space	162	(48.4)
Type of specimen		
Superficial/deep incisional site drainage	102	
Organ/space drainage	97	
Sputum	12	
Blood	7	
Respiratory specimen	6	
Other drainage	2	
Tissue	1	
Other	3	
Causative pathogens		
MRSA	64	
MSSA	54	
*Streptococcus* spp.	17	
*Pseudomonas aeruginosa*	14	
*Corynebacterium* spp.	12	
CNS	10	
*Staphylococcus epidermidis*	7	
*Enterococcus faecalis*	6	
*Escherichia coli*	4	
*Klebciella pneumoniae*	4	
Others	26	
Polymicrobial	30	

*CNS* central nervous system, *MRSA* Methicillin-resistant *Staphylococcus aureus, MSSA* methicillin-susceptible *Staphylococcus aureus*, *SSI* surgical site infections

### Classification of departments by the frequency of surgeries

A median of 47.8 (Inter Quartile Range: 11.4–110.4) procedures (the frequency of surgeries per department) were conducted per year. The distribution among the 74 departments is shown in Additional Fig. 1. In total, 38, 16, and 20 departments were classified as the centers of low-, moderate-, and high-frequency of surgeries, respectively. A total of 3086 (11.5%), 5718 (21.3%), and 18,074 (67.2%) THOR procedures were conducted in these departments annually, and the median numbers were 11.8, 68.6, and 145.2, respectively. A comparison of the three department groups (Table 2) revealed statistically significant differences in the number of beds (*P* < 0.0001, Kruskal–Wallis test), teaching hospitals (*P* = 0.0012, Fisher’s exact test), and tertiary hospitals (*P* = 0.0074, chi-square test). All four teaching hospitals were included in the group with a high-frequency of surgeries, which accounted for 20% of the hospitals in this group. Meanwhile, the tertiary hospitals accounted for 13.2, 37.5, and 50% of the hospitals in the groups with low-, moderate-, and high-frequency of surgeries, respectively.

**Table 2:** Classification of departments in terms of frequency of surgeries, and univariate analysis of risk and confounding factors of SSI

		Department groups in terms of average number of all kinds of procedures/year			Univariate analysis against	
					Department group	SSI
	total	< 50/year	50–100/year	> 100/year	*p* value	*p* value
Department, N	74	38	16	20		
Department characteristics						
Mean of number of beds	409.5	315	449.4	557	< 0.0001	
Number of teaching hospitals	4	0	0	4	0.0012	
Number of tertiary hospitals	21	5	6	10	0.0074	
Number of cancer centers	3	0	1	2	0.11	
Thoracic surgery						
Total number (from 2014 to 2018)	26,878	3086	5718	18,074		
Number of surgical site infections	335	43	60	232		
Rate of surgical site infections (%)	1.25	1.39	1.05	1.28		
Median number of procedures / year	72.6	11.8	68.6	145.2		
Open thoracic surgery						
Procedures	5724	841	1051	3832		
Number of surgical site infections	100	10	25	65		
Rate of surgical site infections (%)	1.75	1.12	2.38	1.7		
Video-assisted thoracic surgery (VATS)					< 0.0001	0.0001
Procedures	21,154	2245	4667	14,242		
Number of surgical site infections	235	33	35	167		
Rate of surgical site infections (%)	1.11	1.47	0.75	1.17		
Age (median, IQR)	68 (55–74)	63 (27–73)	68 (57–75)	68 (59–74)	< 0.0001	0.06
Sex					< 0.0001	< 0.0001
Female	7345 (34.7%)	582 (25.9%)	1575 (33.7%)	5188 (36.4%)		
Male	13,809 (65.3%)	1663 (74.1%)	3092 (66.3%)	9054 (63.6%)		
Emergency operation					< 0.0001	0.605
Yes	1187 (5.6%)	264 (11.8%)	152 (3.3%)	771 (5.4%)		
No	19,967 (94.4%)	1981 (88.2%)	4515 (96.7%)	13,471 (94.6%)		
NNIS risk index					< 0.0001	< 0.0001
0	14,160 (66.9%)	1601 (71.3%)	2415 (51.7%)	10,144 (71.2%)		
1	6343 (30.0%)	565 (25.2%)	2001 (42.9%)	3777 (26.5%)		
2,3	651 (3.1%)	78 (3.5%)	251 (5.4%)	321 (2.3%)		

*NNIS* National Nosocomial Infections Surveillance System, *SSI* surgical site infections

### Univariate analysis of risk and confounding factors of SSI

As indicated above, VATS accounted for the majority (78.7%) of THOR procedures. We also found a statistically significant difference in the proportion of procedures using VATS among the three department groups (72.7, 81.6, and 78.8%, *P* < 0.0001, chi-square test) and in the rate of SSI between VATS and other procedures (1.11 and 1.75%, *P* = 0.0001, chi-square test). These results indicated that VATS was a confounding factor significantly associated with both the department groups and the rate of SSI. To control for its effect in examining the association between them, we confined the procedures using VATS in the following analyses.

Descriptive statistics calculated from the data of the VATS procedures are shown in the middle and end of Table 2. In total, 2245, 4667, and 14,242 VATS procedures were performed in the groups with low-, moderate-, and high-frequency of surgeries, respectively. Meanwhile, 33 (1.47%), 35 (0.75%), and 167 (1.17%) SSIs occurred in the above groups, respectively. There were significant differences in age (*P* < 0.0001, Kruskal-Wallis test), number of male patients (*P* < 0.0001, chi-square test), emergency operations (*P* < 0.0001, chi-square test), and NNIS risk index (*P* < 0.0001, chi-square test) among the groups (Table 2).

Regarding risk factors for SSIs, we found a significantly higher risk in men (*P* < 0.0001, chi-square test, 1.33% in men, vs. 0.69% in women) and in those with an NNIS risk index of 2–3 (*P* < 0.0001, chi-square test; risk index 0, 0.71%; 1, 1.80%; and 2–3, 3.80%]). This indicated that sex and the NNIS risk index, in addition to VATS, were the confounding factors.

### Multiple logistic regression analysis stratified by the NNIS risk index

We conducted multiple logistic regression analyses controlling for confounding factors. Specifically, sex was added as a covariate, and the data were stratified by the NNIS risk index, enabling estimation of the OR for each NNIS risk index. The results revealed a statistically significant association between the department groups and SSI in the strata of NNIS risk indices 1 and 2–3 (Table 3), but not in the stratum of NNIS risk index 0. In the strata of NNIS risk indices 1 and 2–3, the risk of SSI was always the lowest in the group with a moderate-frequency of surgeries. When the group with a moderate-frequency of surgeries was used as a reference, both the groups with low- (OR: 2.48 [95% CI 1.20–5.13], *P* = 0.0143) and high- (OR: 2.43 [95% CI 1.44–4.11], *P* = 0.0009) frequency of surgeries were significantly positively associated with SSI in the stratum of NNIS risk index 1. A similar association was found between SSI and male sex (OR: 2.13 [95% CI 1.31–3.48] *P* = 0.0024). In the stratum of NNIS risk indices 2 and 3, only the group with a low frequency of surgeries was significantly positively associated with SSI (OR: 4.83, 95% CI: 1.47–15.93, *P* = 0.0095). (Table 3).

**Table 3:** Multiple logistic regression analysis stratified by the NNIS risk index

	OR	95% CI	*p* value
NNIS risk index 1 group			
Male sex	2.13	1.31–3.48	0.0024
Age	1.02	1.01–1.03	0.0021
< 50/year	2.48	1.20–5.13	0.0143
50–100/year	Reference	Reference	Reference
> 100/year	2.43	1.44–4.11	0.0009
NNIS risk indices 2–3 group			
Male sex			0.063
Age			0.65
< 50/year	4.83	1.47–15.93	0.0095
50–100/year	Reference	Reference	Reference
> 100/year			0.21

*NNIS* National Nosocomial Infections Surveillance System

## Discussion

The association between the frequency of surgeries and the incidence of SSIs in VATS is yet to be clarified. Our study revealed that low- and high-frequency of surgeries were associated with significantly higher rates of SSIs than moderate-frequency of surgeries in the cases with NNIS risk index 1. Meanwhile, in the cases of NNIS risk index 2 or 3, a low-frequency of surgeries was associated with significantly higher rate of SSIs. To the best of our knowledge, this is the first study to investigate the relationship between the frequency of surgeries and SSIs in THOR procedures based on a large national surveillance database. After focusing on VATS, which accounted for most procedures and was found to be a confounding factor, we found a significant association between the stratum of NNIS risk index 1 and that of NNIS risk indices 2–3.

The frequency of surgeries was classified using the total number of procedures, including VATS and open thoracic surgery. Further, similar results were obtained if only the annual number of VATS procedures was used, with the multiple logistic regression analysis adjusted for sex and stratified by NNIS risk index (Additional Table 1). A high-frequency of surgeries was significantly positively associated with SSIs in the stratum of NNIS risk index 1 (OR: 1.94 [95% CI 1.20–3.15], *P* = 0.0066). In the stratum of NNIS risk indices 2 and 3, only the low-frequency of surgeries was significantly positively associated with SSI (OR: 3.76, 95% CI: 1.25–11.26, *P* = 0.0181).

Superficial and deep incisional infections accounted for 51.6% of all SSIs, indicating that both VATS and open thoracic surgeries influenced the incidence of SSI. Okada et al. reported the effectiveness of hybrid video-assisted thoracic surgery, a combination of VATS and open thoracic surgery (5-year overall survival: 89.8%, 5-year disease-free survival: 84.7%) [24], supporting the importance of both VATS and thoracic surgery in general. Therefore, we considered that the result using the average number of all kinds of procedures annually as a measure of the frequency of a department’s surgeries would be more accurate than that confined to VATS.

In general, it is expected that a higher frequency of surgeries would be associated with a lower risk of SSI. This has been consistently shown in several previous studies that examined the association between frequency of surgeries and SSI rates in other types of surgeries [23, 25]. However, our study revealed that the group with the moderate- frequency of surgeries had the lowest risk of SSIs in the NNIS risk index 1 stratum. To our best knowledge, this is the first study to report such a finding. This result could be explained by the significantly higher proportion of the teaching hospitals and tertiary hospitals in the high-frequency of surgeries group than in the other groups. Previously, Mu et al. analyzed the NHSN SSI surveillance data, including thoracic surgeries, and reported that a bed number greater than 500 was associated with a significantly higher SSI risk after adjusting for other risk factors [9]. The present study is consistent with these findings, but further revealed that hospitals conducting a high frequency of surgeries had a significantly higher number of beds and were more likely to be teaching and tertiary hospitals than others (Table 2). Teaching and tertiary hospitals are responsible for training doctors, which could inadvertently cause a higher incidence of SSI in the NNIS risk index 1. Another possible explanation is the higher proportion of complicated cases or organized infection control teams that can report SSI without omissions, although such data are not available.

This result is somewhat consistent with those reported by Furuya-Kanamori et al., who showed that the groups with a high-frequency of surgeries tended to have a higher risk of SSI than the groups with a low-frequency of surgeries in colorectal surgeries, although they did not stratify and control for the risk index [16]. Meanwhile, in the stratum of NNIS risk indices 2–3, there was no significant difference between the middle and high-frequency of surgeries groups, perhaps because experienced instructors, rather than inexperienced ones, could be responsible for the operations of complicated cases in the group with the high-frequency of surgeries. Umana-Pizano et al. reported a difference in outcomes following acute type A dissection between surgeons with low-frequency of surgeries and those with high-frequency of surgeries in high-frequency of surgeries centers [26]. To the best of our knowledge, all studies that examined the relationship between surgery and the rate of SSIs were reported from high-income countries. There was no SSI surveillance system in low- and middle-income countries [18]. Anderson et al. reported a relationship between the frequency of surgeries and postoperative mortality rate in a single center and the difficulty of collecting data in low-income countries [27].

The other risk factor of SSI in NNIS risk index 1 was the male sex. Based on analyses using the German national nosocomial surveillance system (OP-KISS), Aghdassi et al. reported that the male sex was one of the risk factors for SSIs in orthopedics, traumatology, and abdominal surgeries. Meanwhile, the female sex was one of the risk factors for SSIs in heart and vascular surgeries [19]. Schroder et al. reported that the male sex was one of the risk factors for SSIs in hip prosthesis (OR: 1.28, 95% CI: 1.11–1.49, *P* = 0.001) and colon surgeries (OR: 1.16, 95% CI: 1.04–1.29, *P* < 0.001) [28]. However, no previous study has reported its positive association with SSI in THOR procedures. This association could be because smoking is more frequent among men than among women in Japan [29], and is a factor affecting SSI [30].

The surveillance data we analyzed did not include detailed information concerning the comorbidities and clinical background (e.g., Brinkman index) of each patient. Despite this limitation, we utilized the large dataset of 26,878 THOR procedures collected from 74 hospitals (departments) across Japan. This number was almost eight-fold higher than that collected (3370 procedures) for 12 years and analyzed in a similar study by Cvijanovic et al. [8] Although they evaluated the risk factors for SSIs in THOR, they did not consider the frequency of surgeries in the analysis. In their study, SSIs occurred in 6.08% of the 3370 procedures and 2.14% of 700 VATS procedures. The large dataset enabled us to elucidate the effect of the frequency of surgeries on SSI stratified by the NNIS risk index. Further, our analysis of the SSI surveillance data focusing on the THOR procedures quantified the effect of the frequency of surgeries per department on SSIs after carefully adjusting for confounding factors, including VATS.

## Conclusion

In conclusion, in the stratum of NNIS risk index 1, SSI incidence was the lowest in the departments with a moderate frequency of surgeries. Meanwhile, in the stratum of NNIS risk indices 2–3, SSI incidence was the lowest in the departments with moderate- and high-frequency of surgeries. These results suggest that for departments with a low-frequency of surgeries, an increase in the frequency of surgeries to > 50 per department per year potentially leads to a decrease in the incidence of SSIs. This would occur through an increase in the experience of surgeons in the departments and contribute to the improvement of VATS outcomes in thoracic surgeries.

## Supplementary Information

**Additional file 1: Figure 1.** Average number of annual procedures per department of 74 hospitals. The vertical axis shows the number of procedures, and the horizontal axis shows the number of hospitals.

**Additional file 2: Table 1.** Multiple logistic regression analysis of risk factors of THOR, including VATS.

## Data Availability

The datasets generated and analyzed during the current study are available at [https://figshare.com/s/09227b8064aee2e8881c].

## Abbreviations

CI: Confidence interval
JANIS: Japan Nosocomial Infections Surveillance
NNIS: National Nosocomial Infections Surveillance System
ORs: Odds ratios
SSIs: Surgical site infections
THOR: Thoracic surgeries
VATS: Video-assisted thoracic surgeries
