# Supplementary material for: Association between the frequency of surgeries for video-assisted thoracic surgery and the incidence of consequent surgical site infections: a retrospective observational study based on national surveillance data
Source: BMC Infect Dis. 2021 Apr 17;21:363. doi: 10.1186/s12879-021-06050-6 (PMC8052810; doi:10.1186/s12879-021-06050-6)
Supplement: Supplementary file 1 — Additional file 1: Figure 1. Average number of annual procedures per department of 74 hospitals. The vertical axis shows the number of procedures, and the horizontal axis shows the number of hospitals. [file 12879_2021_6050_MOESM1_ESM.pptx]

## Slide 1
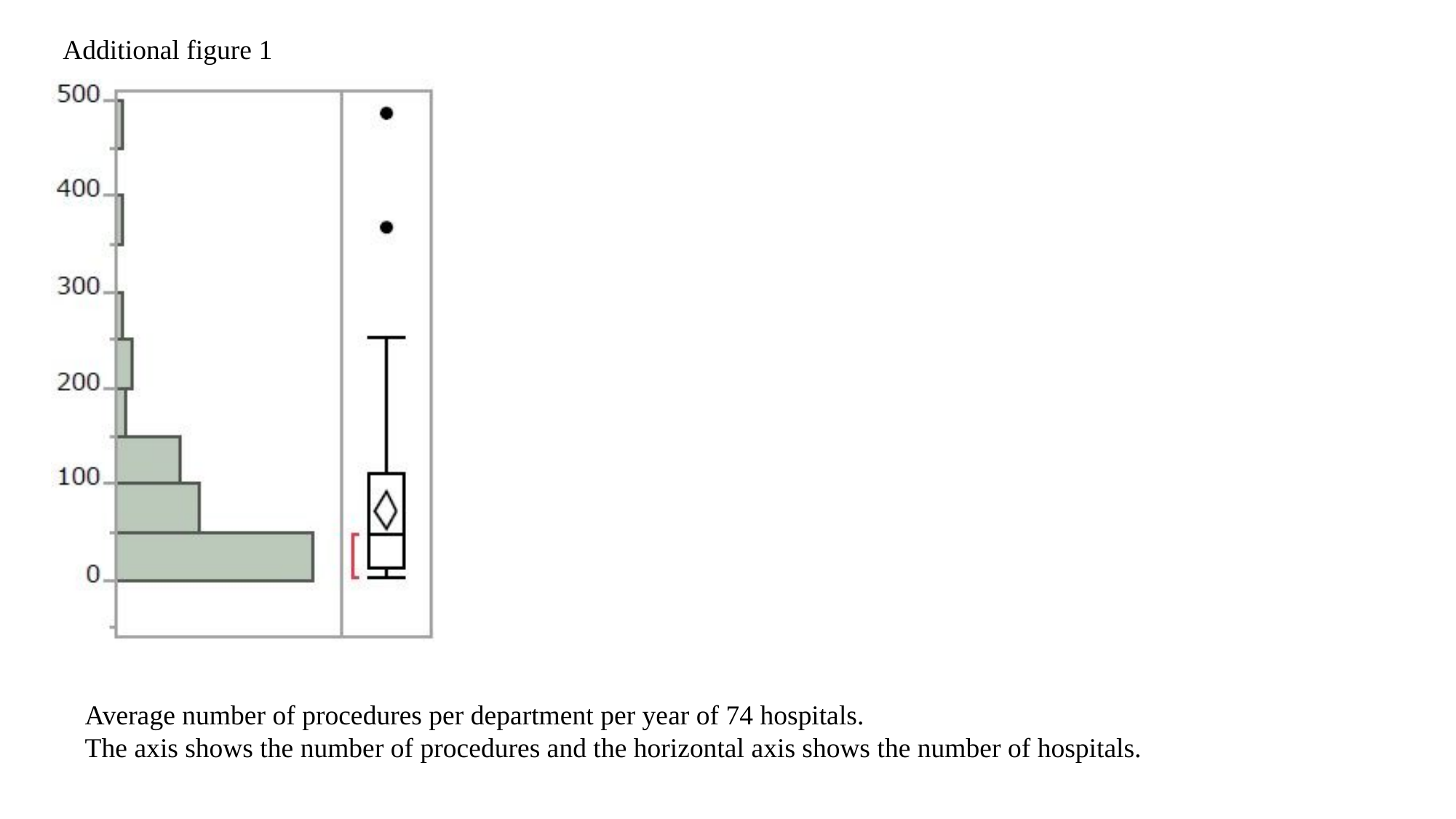

Additional figure 1
Average number of procedures per department per year of 74 hospitals.
The axis shows the number of procedures and the horizontal axis shows the number of hospitals.
