# Supplementary material for: Association between the frequency of surgeries for video-assisted thoracic surgery and the incidence of consequent surgical site infections: a retrospective observational study based on national surveillance data
Source: BMC Infect Dis. 2021 Apr 17;21:363. doi: 10.1186/s12879-021-06050-6 (PMC8052810; doi:10.1186/s12879-021-06050-6)
Supplement: Supplementary file 2 — Additional file 2: Table 1. Multiple logistic regression analysis of risk factors of THOR, including VATS. [file 12879_2021_6050_MOESM2_ESM.docx]

Table S1. Multiple logistic regression analysis of risk factors of THOR including VATS

|  | OR | 95% CI | p value |
| --- | --- | --- | --- |
| NNIS Risk Index 1 group |  |  |  |
| Male sex | 2.18 | 1.34-3.55 | 0.0018 |
| Age | 1.02 | 1.01-1.04 | 0.0010 |
| <50/year |  |  | 0.39 |
| 50-100/year | Reference | Reference | Reference |
| >100/year | 1.94 | 1.20-3.15 | 0.0066 |
|  |  |  |  |
| NNIS Risk Index 2-3 group |  |  |  |
| Male sex |  |  | 0.0762 |
| Age |  |  | 0.68 |
| <50/year | 3.76 | 1.25-11.26 | 0.0181 |
| 50-100/year | Reference | Reference | Reference |
| >100/year |  |  | 0.25 |
